# Systematic Review: Evaluating the Effect of Lipid-Lowering Therapy on Lipoprotein and Lipid Values

**DOI:** 10.1007/s10557-013-6477-6

**Published:** 2013-07-28

**Authors:** Robert S. Rosenson, James A. Underberg

**Affiliations:** 1 Icahn School of Medicine at Mount Sinai, New York, NY 10029 USA; 2 Department of Medicine, New York University Langone Medical Center, New York, NY 10016 USA

**Keywords:** Low density lipoprotein-particle (LDL-P), Nuclear magnetic resonance (NMR)-based LDL-P, Systematic review, Lipid lowering pharmacotherapy, Cardiovascular disease

## Abstract

**Purpose:**

This systematic review was performed to summarize published experience using low density lipoprotein particle number (LDL-P) to monitor the efficacy of lipid-lowering pharmacotherapies.

**Methods:**

Studies were identified from a literature search of MEDLINE (January 1, 2000 – June 30, 2012); and abstract searches of select conferences. All accepted studies reported mean (or median) nuclear magnetic resonance (NMR)-based LDL-P values for at least 10 subjects receiving lipid lowering pharmacotherapy.

**Results:**

Searches revealed 36 studies (with 61 treatment arms) in which LDL-P measurements were reported pre- and post-treatment. Most studies also reported changes in low-density lipoprotein cholesterol (LDL-C), but fewer studies reported changes in apolipoprotein B (apoB)(*n* = 20) and non-HDL-C (*n* = 28). Treatments included statins (22 arms/15 studies), fibrates (7 arms/7studies), niacin (7 arms/6 studies), bile acid sequestrants (5 arms/2 studies), an anti-apoB oligonucleotide (2 arms/2 studies), combination therapies (8 arms/6 studies), anti-diabetics (5 arms/4 studies), and, other treatments (5 arms/2 studies). Lipid-lowering pharmacotherapy resulted in reductions in mean LDL-P in all but two studies. In several statin studies, the percent reductions in LDL-P were smaller than reductions in LDL-C, comparable changes were reported when LDL-P and apoB, were reported.

**Conclusions:**

Study-level data from this systemic review establish that different lipid lowering agents can lead to discordance between LDL-P and LDL-C, therefore, basing LDL-lowering therapy only on the achievement of cholesterol goals may result in a treatment gap. Therefore, the use of LDL-P for monitoring lipid-lowering therapy, particularly for statins, can provide a more accurate assessment of residual cardiovascular risk.

## Introduction

The association between low-density lipoprotein (LDL) particles and cardiovascular disease (CVD) is well established [1, 2]. Evidence from lipid lowering clinical trials demonstrates that LDL lowering therapy reduces cardiovascular events [3]. Currently, there are number of methods available commercially to measure LDL. LDL cholesterol (LDL-C) and non-high-density lipoprotein cholesterol (non-HDL-C) rely on the cholesterol content of the lipoprotein to measure the efficacy of LDL-lowering therapy whereas LDL particle (LDL-P) number and apolipoprotein B (apoB) levels are used to measure the actual quantity of LDL [3].

LDL particles contain a core of lipid, predominantly cholesterol esters and a minor amount of triglycerides, surrounded by a shell of phospholipid on which the major surface protein is apoB [3]. LDL-C measures the amount of cholesterol packaged in the LDL particle and has served as the basis for the assessment and management of CVD risk for many years; however, a number of recent studies have shown that CVD risk may be better predicted by measuring the concentration of LDL-P [1, 2, 4, 5]. The cholesterol and triglyceride content of LDL particles vary widely among individuals and can change over time as a result of lifestyle changes, metabolic disease, and lipid-lowering therapy. It has been reported that statins, estrogen replacement therapy, and a low fat/high carbohydrate diet lower the LDL-C content in LDL particles more than they lower the LDL-P concentration, while fibrates, nicotinic acid, exercise and a low carbohydrate diet lower LDL-P concentration more than they lower LDL-C content [3]. Thus, reliance on LDL-C rather than LDL-P as a measure of CVD risk may result in suboptimal management of LDL-related risk in certain individuals.

In 2011, the National Lipid Association (NLA) held a consensus conference focused on the use of inflammatory markers and advanced lipoprotein testing to improve CVD risk assessment and management of lipid-lowering therapy. Their consensus statement concluded that measurement of LDL-P would be a “reasonable measure” to incorporate into treatment decisions for many patients at intermediate and high risk treated to LDL-C and non-HDL-C goals in order to evaluate the adequacy of LDL lowering therapy [6]. However, the panel noted: “additional research is needed to more clearly define settings in which a policy of treating to LDL-P goals might produce more favorable outcomes than the alternative of treating to LDL-C and non-HDL-C goals”.

To assess the utilization of LDL-P measurements for the management of lipid-lowering therapy, a systematic review of published studies was conducted which focused on study level comparisons of treatment-related changes in LDL-P versus other lipid parameters, including LDL-C, non-HDL-C, and apoB. We report here study-level correlations between treatment-related changes in lipid and lipoprotein parameters and CVD progression.

## Materials and Methods

### Literature Search and Selection

An electronic search of PubMed was performed using the following search strategy, where “MeSH” indicates Medical Subject Headings:

1. Lipoproteins, LDL [MeSH] OR low-density lipoprotein particle OR LDL OR particle test OR LDL-C OR LDL-P
2. Magnetic Resonance Spectroscopy [MeSH] OR NMR OR nuclear magnetic resonance
3. #1 AND #2; Limits: English, Human, January 1, 2000 –July 30, 2012, NOT case reports, letters, editorials, reviews, new articles

Note that articles available as Epub ahead of print before the search cut-off date were considered eligible for inclusion; even if the eventual print publication date was after July 30, 2012. The electronic search was supplemented by manual searches of 2010 – 2012 abstracts for the annual meetings of the American College of Cardiology, and American Diabetes Association, National Lipid Association and American Heart Association (2010 and 2011 conferences only), (screened first by title and then by review of the complete abstract) and a limited manual search of reference lists from accepted studies and recent reviews.

Studies of any design (prospective or retrospective, randomized or non-randomized) were selected for review if they reported mean (or median) magnetic resonance-based LDL-P values for at least 10 patients prior to and after receiving lipid-lowering pharmacotherapy; studies reporting LDL-P changes only after nutritional supplements or lifestyle changes (exercise, diet, smoking cessation) were not included. Screening was performed by a single reviewer. Multiple publications of the same or overlapping series of patients were identified and grouped together as a “kinned” citation; the parent study was most often the most recent publication. Data from kinned studies were counted only once to avoid the double-counting of patients.

### Extraction of Study-Level Variables

Data were extracted by two independent reviewers and discrepancies resolved by consensus conference. The following data elements were sought from each accepted study: study characteristics, including PMID number, first author, geographic location by country, publication year, study type [case series, cohort, Randomized Control Trial (RCT)], and lipid lowering treatment [generic drug name, dose, and treatment duration; population characteristics, including underlying condition(s) requiring lipid-lowering therapy, number of subjects enrolled, mean/median age, gender distribution, racial distribution, and number of subjects with CVD, present family history of CVD, diabetes, metabolic syndrome, smoking, hypertension (blood pressure >140/90 mm Hg or on medication), obesity (body mass index ≥ 35 kg/m^3^); and LDL-P, and if available, LDL-C, HDL-C, non-HDL-C, and apoB prior to and after lipid-lowering treatment; and if reported, CVD outcomes and changes in atherosclerotic plaque burden (carotid intima media thickness (CIMT), coronary artery calcium (CAC) score) and/or correlations of changes in LDL-P and CVD outcome and changes in atherosclerotic plaque burden.

On-treatment results for LDL-C, apoB and non–HDL-C are expressed in milligrams per deciliter and LDL-P in nanomoles per liter. In addition, each parameter is expressed in terms of population percentiles based on data from the Framingham Offspring Study [7].

Study, patient, and test-level data were summarized using general descriptive statistics (means, standard deviations, ranges, counts) and subgroup analyses were performed by type of lipid-lowering therapy.

## Results

### Search Yields

**Fig 1:**
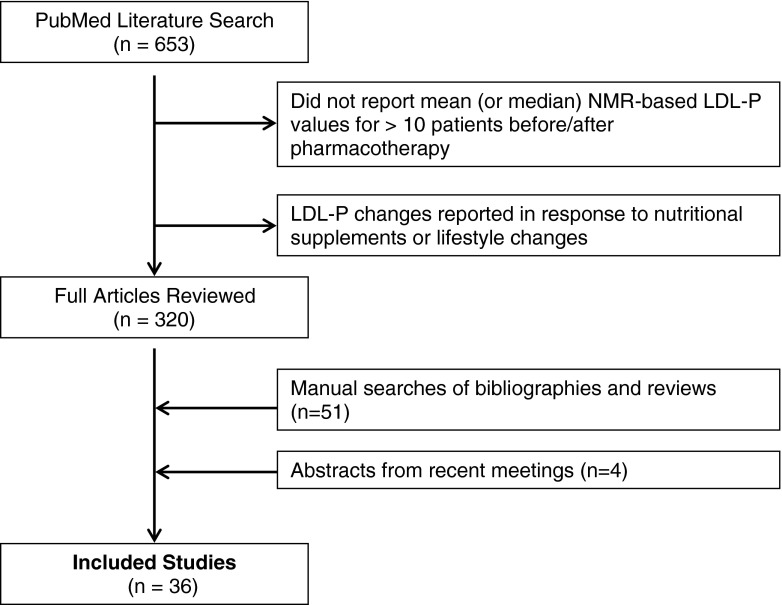
Study flow diagram

From the PubMed search, a total of 653 citations were screened and 320 full publications were retrieved for further review (Fig. 1). Manual searches of bibliographies and recent reviews yielded 51 additional studies, and manual searches of annual meeting abstracts yielded 4 additional studies (reported only as abstracts) for further review. From these, a total of 36 primary studies reported treatment-induced changes in mean (or median) NMR-derived LDL-P values following lipid-lowering pharmacotherapy. These studies served as the basis for this analysis. In addition to LDL-P, most accepted studies also reported changes in mean (or median) LDL-C (31 studies), non-HDL-C (28 studies) and/or apoB (20 studies). Baseline concentrations of LDL-P have been reported for the Medical Research Council/British Heart Foundation Heart Protection Study, one of the largest randomized control trials of statin therapy to date, however LDL-P was not measured following treatment so this study was not included.

Study and treatment characteristics are summarized in Table 1. Mean (or median) nuclear magnetic resonance (NMR)-based LDL-P values were reported pre- and post-treatments with statins (1876 subjects in 22 treatment arms), fibrates (788 subjects in 7 arms), niacin (580 subjects in 7 arms), bile acid sequestrants (139 subjects in 5 arms), combination therapies (1342 subjects in 8 treatment arms), anti-apoB oligonucleotide therapy (104 subjects in 2 treatment arms), anti-diabetic therapies (848 subjects in 5 treatment arms) and other treatments (717 subjects in 5 treatment arms). Most studies were short-term (median of 3 months per treatment arm), randomized controlled trials (28), and designed to demonstrate lipid lowering for primary prevention (27/36). Only four studies reported CVD outcomes.

**Table 1:** Summary of study and patient characteristics

Parameter	Total studies^a^	Combination therapy	Type of pharmacotherapy^b^				
			Statins	Fibrates	Niacin	Bile acid Sequestrant	Anti-diabetic
Number of studies	36	6	15	7	6	2	4
Number of treatment arms	61	8	22	7	7	5	5
Number of patients	6394	1342	1876	788	580	139	848
Study type:							
Cohort/other	8	0	3	3	1	1	0
RCT	28	6	12	4	5	1	4
Study population							
Primary prevention	27	2	11	4	4	2	3
Secondary prevention	9	1	4	1	2	0	0
Median treatment per arm (months)	3	4	3	3	3	1.5	5
Range (months)	1 – 36	1 –12	1–12	1 – 12	3 – 12	1.0 – 1.5	3 – 6
Arms reporting pre-post changes in							
LDL-P	36	8	22	7	7	5	5
LDL-C	31	6	17	5	6	5	5
apoB	20	6	14	3	4	1	2
Non-HDL-C	28	6	16	5	5	1	5
Studies reporting CVD outcomes							
CVD events	1	0	0	1	0	0	0
Change in atherosclerotic plaque	3	0	1	1	0	0	0

*RCT* Randomized Control Trial; *LDL-P* low-density lipoprotein particle number; *LDL-C* low-density lipoprotein cholesterol; *apoB* apolipoproteinB; *Non-HDL-C* non-high-density lipoprotein cholesterol; *CVD* cardiovascular disease

^a^some studies had treatment arms for more than one type of pharmacotherapy;

^b^excludes two studies of an anti-apoB oligonucleotide and two studies of statin therapy noted only as aggressive or standard treatment and pravastatin and dalcetrapib at 300, 600 and 900 mg;

### Treatment-Induced Changes in Lipid Parameters

Results from 36 studies are summarized in Tables 2, 3, 4, 5, 6, 7, 8, and 9. Reductions in LDL-P were observed with all treatments except for two (one fibrate, one anti-diabetic). Similarly, LDL-C was reduced with all treatments except for the fibrates, anti-diabetic therapy and the cholesteryl ester transfer protein (CETP) inhibitor dalcetrapib. Of the marketed lipid-lowering monotherapies studied, statins produced the greatest reductions in LDL-P and LDL-C, followed by niacin, fibrates and bile acid sequestrants. In addition, the combination of statins with other agents yielded an even greater effect on LDL-P and LDL-C. The effects of each type of lipid-lowering or anti-diabetic therapy on LDL-P and LDL-C are discussed below in terms of average results across all studies within each drug class, not accounting for differences in the agent, dose, time period, size of the treatment group or characteristics of the population at baseline.

**Table 2:** Effects of statin therapy on LDL-related parameters^a^

Trial	Treatment	n	time (mo)	LDL-C				LDL-P				Non HDL-C				ApoB			
				baseline	treatment	% dec	%tile	baseline	treatment	% dec	%tile	baseline	treatment	% dec	%tile	baseline	treatment	% dec	%tile
Airan-Javia[20]	S20	25	12	102	99	3	23	1,203	1,112	8	25	133	133	0	34	82	84	−2	33
[RCT;2°;carotid athero]	S80	24	12	107	81	24	7	1,211	960	21	14	137	103	25	12	88	77	13	24
PRINCE[15]	P40	256	3	144	110	24	32	1,540	1,265	18	36	192 ^b^	147 ^b^	23	45	NR	NR	–	–
[RCT;1°;no known CAD]																			
Chan[21]	S20	35	3	NR	NR	–	–	2,194	1,553	29	57	NR	NR	–	–	NR	NR	–	–
[RCT;1°;no known CAD]																			
Ikewaki[8]	A10	26	1	195	111	43	33	1,802	1,086	40	23	226 ^b^	134 ^b^	41	35	137	90	34	40
[Cohort;1°;hyperchol]																			
Jones[9]	LDS (S20, A20 or R10)	153	3	NR	NR	–	–	1,969	1,255	36	35	NR	NR	–	–	NR	NR	–	
[RCT;1°;mixed dyslipid]	MDS (S40, A40 or R20)	170	3	NR	NR	–	–	1,982	1,159	42	28	NR	NR	–	–	NR	NR	–	
McKenney[10, 32]	A10	55	3	171	117	32	38	2,562	1,757	31	71	238 ^b^	160 ^b^	33	55	156	109	30	64
[RCT;1°;dyslipid]																			
Maki[11]	A10	122	1	146	98	33	22	1,946	1,322	32	40	216	142	34	41	152	102	33	55
[RCT;1°;mixed dyslipid]																			
Miller[22]	S 40	20	1.5	166	88	47	13	2,297	1,456	37	50	227 ^b^	136 ^b^	40	37	144	101	30	54
[RCT;1°;mixed dyslipid]	S 80	20	1.5	166	83	50	9	2,297	1,349	41	42	227 ^b^	122 ^b^	46	26	144	86	40	35
PLAC-1[16]	P 20/40	154	6	162	120	26	41	1,908	1,432	25	48	190 ^b^	144 ^b^	24	43	NR	NR	–	–
[RCT;2°;CHD]																			
COMETS[12]	A 10/20	91	3	170	95	44	19	1,869	1,234	34	34	205 ^b^	119 ^b^	43	24	161	101	37	54
[RCT;1°;met syn]	R 10/20	166	3	166	83	50	9	1,962	1,216	38	32	205 ^b^	107 ^b^	48	15	160	93	42	44
Schaefer[13]	A 40	27	1	161	89	45	14	2,454	1,399	43	46	224	121	46	25	174	104	40	58
[Cohort;2°;CHD]	P 40	22	1	187	142	24	60	2,454	1,914	22	82	242	184	24	73	185	155	16	>95
	S 40	25	1	205	125	39	45	2,454	1,571	36	58	262	160	39	55	185	139	25	>95
CARDS[14]	A 10	69	6	134	71	47	2	1,572	1,065	32	22	167	84	50	<2	NR	NR	–	–
[RCT;2°;T2D, IHD]																			
Sponseller [17]	Pit 4	164	3	164	NR	–	–	1,758	1,238	30	34	196 ^b^	NR	–	–	126	95	25	46
[RCT;1°;dyslipid]	P 40	164	3	165	NR	–	–	1,740	1,368	21	43	199 ^b^	NR	–	–	128	106	17	60
Stein [18]	P 40	20	2	134	107	20	30	1,756	1,384	21	44	179	139	22	39	NR	NR	–	–
[Crossover;1°;HIV]																			
van der Graaf [19]	P 20/40	68	12	238	181	24	94	1,783	1,508	15	53	255^b^	NR	–	–	NR	NR	–	–
[RCT;1°;FH]																			
		Total n	Mean time	Mean LDL-C				Mean LDL-P				Mean non-HDL-C				Mean ApoB			
				baseline	treatment	% dec	%tile	baseline	treatment	% dec	%tile	baseline	treatment	% dec	%tile	baseline	treatment	% dec	%tile
		1876	4	162 +32	106 ± 27	34 ± 13	29 ± 23	1942 ± 380	1346 ± 226	30 ± 10	42 ± 16	206 ± 35	133 ± 24	34 ± 13	35 ± 18	144 ± 31	103 ± 21	27 ± 13	54 ± 21

Doses in mg/day; *LDS* Low-dose statin; *MDS* Moderate-dose statin; *S* Simvastatin; *P* Pravastatin; *A* Atorvastatin; *R* Rosuvastatin; *Pit* Pitavastatin; *carotid athero* carotid atherosclerosis; *CAD* coronary artery disease; *hyperchol* hypercholesterolemia; *mixed dyslipid* mixed dyslipidemia; *dyslipid* dyslipidemia; *CHD* coronary heart disease; *met syn* metabolic syndrome; *T2D* type 2 diabetes; *IHD* ischaemic heart disease; *HIV* human immunodeficiency virus; *FH* familial hypercholesterolaemia

^a^Month defined as 4 weeks, *LDL* low-density lipoprotein, *LDL-C* low-density lipoprotein cholesterol, *LDL-P* low-density lipoprotein particle number, *Non HDL-C* non-high-density lipoprotein cholesterol, *ApoB* apolipoprotein B, *% dec* percent decrease, *%tile* Percentile of Framingham population; *NR* not reported, *RCT* Randomized Control Trial, *1°* primary intervention, *2°* secondary intervention

^b^calculated from values given in the Trial

**Table 3:** Effects of fibrate therapy on LDL-related parameters^a^

Trial	Treatment	n	time (mo)	LDL-C				LDL-P				Non-HDL-C				ApoB			
				baseline	treatment	% dec	%tile	baseline	treatment	% dec	%tile	baseline	treatment	% dec	%tile	baseline	treatment	% dec	%tile
Ayaori [25]	Bz 400	14	12	123	136	−11	55	1716	1765	−3	72	207^b^	167^b^	19	60	NR	NR	–	–
[Cohort;1°; dyslipid]																			
Chan [21]	F 200	35	3	NR	NR	–	–	2156	1794	17	74	NR	NR	–	–	NR	NR	–	–
[RCT;1°; no known CAD]																			
Ikewaki [23]	F 200	20	2	99	100	−1	23	1567	1396	11	45	191^b^	161^b^	16	55	118	107	9	61
[Cohort;1°; hyperTG]																			
Ikewaki [26]	Bz 400	24	1	124	139	−12	57	1722	1643	5	63	198^b^	172^b^	13	64	117	112	4	68
[Cohort;1°; hyperTG]																			
Jones [9]	F 135	155	3	NR	NR	–	–	2034	1625	20	62	NR	NR	–	–	NR	NR	–	–
[RCT;1°; mixed dyslipid]																			
VA-HIT [4]	G 1200	515	7	112	115	−3	36	1352	1290	5	38	144	137	5	37	95	89	6	39
[RCT;2°;CHD]																			
Rosenson[24, 55]	F 160	25	3	139	131	6	50	1737	1421	18	47	188	157	16	52	NR	NR	–	–
[RCT;1°; metabolic syndrome hyperTG]																			
		Total n	Mean time	Mean LDL-C				Mean LDL-P				Mean non-HDL-C				Mean ApoB			
				baseline	treatment	% dec	%tile	baseline	treatment	% dec	%tile	baseline	treatment	% dec	%tile	baseline	treatment	% dec	%tile
		788	4	119 ± 12	124 ± 17	−6 ± 5	44 ± 15	1746 ± 272	1562 ± 195	10 ± 9	57 ± 14	186 ± 24	159 ± 13	14 ± 6	54 ± 10	110 ± 13	103 ± 12	6 ± 3	56 ± 15

Doses in mg/day; *Bz* Bezafibrate; *F* Fenofibrate; *G* Gemfibrozil; *dyslipid* dyslipidemia; *CAD* coronary artery disease; *hyper TG* hypertriglyceridemia; *mixed dyslipid* mixed dyslipidemia; *CHD* coronary heart disease

^a^Month defined as 4 weeks, *LDL* low-density lipoprotein, *LDL-C* low-density lipoprotein cholesterol, *LDL-P* low-density lipoprotein particle number, *Non HDL-C* non-high-density lipoprotein cholesterol, *ApoB* apolipoprotein B, *% dec* percent decrease, *%tile* Percentile of Framingham population; *NR* not reported, *RCT* Randomized Control Trial, *1°* primary intervention, *2°* secondary intervention

^b^calculated from values given in the Trial

**Table 4:** Effects of niacin therapy on LDL-related parameters^a^

Trial	Treatment	n	time (mo)	LDL-C				LDL-P				Non-HDL-C				ApoB			
				baseline	treatment	% dec	%tile	baseline	treatment	% dec	%tile	baseline	treatment	% dec	%tile	baseline	treatment	% dec	%tile
Bays [31, 33]	ERN/LRPT 1 g	303	9	85	70	18	2	1126	950	16	13	115^c^	95^c^	17	6	90	75	17	21
[RCT;1°;T2D]																			
Dube [27]	ERN 0.5-2 g	32	12	NR	NR	–	–	1780	1757	1	71	217	197	9	83	132	119	10	76
[Cohort;1°;HIV]																			
Jafri [28]	ERN 1 g	27	3	76	72	5	2	1033	942	9	13	97^b^	89^b^	8	1	NR	NR	–	–
[RCT;2°;CAD]																			
McKenney [10, 32]	N 3 g	53	3	177	168	5	82	2561	2199	14	>95	242^b^	208^b^	14	91	159	136	14	98
[RCT;1°;dyslipid]																			
Le [29]	N 2 g	124	6	158	125	21	45	1729	1357	22	43	192	148	23	<2	150	120	20	78
[RCT;1°;dyslipid]																			
Morgan [30]	ERN 1 g	21	3	202	190	6	>95	1993	1693	15	67	NR	NR	–	–	NR	NR	–	–
[RCT;1°;dyslipid]	ERN 2 g	20	3	211	181	14	94	2048	1574	23	58	NR	NR	–	–	NR	NR	–	–
		Total n	Mean time	Mean LDL-C				Mean LDL-P				Mean non-HDL-C				Mean ApoB			
				baseline	treatment	% dec	%tile	baseline	treatment	% dec	%tile	baseline	treatment	% dec	%tile	baseline	treatment	% dec	%tile
		580	6	152 ± 58	134 ± 54	12 ± 7	53 ± 44	1753 ± 534	1496 ± 453	14 ± 7	51 ± 29	173 ± 64	147 ± 55	14 ± 6	36 ± 46	133 ± 31	113 ± 26	15 ± 4	68 ± 33

Doses in g/day; *ERN* Extended release niacin; *LRPT* Larpiprant; *N* Niacin; *T2D* type 2 diabetes; *HIV* human immunodeficiency virus; *CAD* coronary artery disease; *dyslipid* dyslipidemia

^a^Month defined as 4 weeks, *LDL* low-density lipoprotein, *LDL-C* low-density lipoprotein cholesterol, *LDL-P* low-density lipoprotein particle number, *Non HDL-C* non-high-density lipoprotein cholesterol, *ApoB* apolipoprotein B, *% dec* percent decrease, *%tile* Percentile of Framingham population; *NR* not reported, *RCT* Randomized Control Trial, *1°* primary intervention, *2°* secondary intervention

^b^calculated from values given in the Trial

^c^calculated using least squares mean

**Table 5:** Effects of bile acid sequestrant therapy on LDL-related parameters^a^

Trial	Treatment	n	time (mo)	LDL-C				LDL-P				Non HDL-C				ApoB			
				baseline	treatment	% dec	%tile	baseline	treatment	% dec	%tile	baseline	treatment	% dec	%tile	baseline	treatment	% dec	%tile
Kishimoto [34]	Cole 3 g	21	1	156	128	18	48	1920	1569	18	58	202^b^	169^b^	16	61	139	121	13	79
[Cohort;1°;dyslipid]																			
Rosenson [25]	Colv 1.5 g	30	1.5	186	179	4	92	2230	2178	2	>95	NR	NR	–	–	NR	NR	–	–
[RCT;1°;dyslipid]	Colv 2.25 g	29	1.5	187	180	4	93	2263	2270	0	>95	NR	NR	–	–	NR	NR	–	–
	Colv 3.0 g	30	1.5	183	164	10	79	2308	2119	8	97	NR	NR	–	–	NR	NR	–	–
	Colv 3.75 g	29	1.5	178	158	11	74	2311	1982	14	87	NR	NR	–	–	NR	NR	–	–
		Total n	Mean time	Mean LDL-C				Mean LDL-P				Mean non-HDL-C				Mean ApoB			
				baseline	treatment	% dec	%tile	baseline	treatment	% dec	%tile	baseline	treatment	% dec	%tile	baseline	treatment	% dec	%tile
		139	1	178 ± 13	162 ± 21	9 ± 6	77 ± 18	2206 ± 164	2024 ± 275	9 ± 8	86 ± 16	202	169	16	61	139	121	13	79

Doses in g/day; *Cole* Colestimide; *Colv* Colesevelam; *dyslipid* dyslipidemia

^a^Month defined as 4 weeks, *LDL* low-density lipoprotein, *LDL-C* low-density lipoprotein cholesterol, *LDL-P* low-density lipoprotein particle number, *Non HDL-C* non-high-density lipoprotein cholesterol, *ApoB* apolipoprotein B, *% dec* percent decrease, *%tile*: Percentile of Framingham population; *NR* not reported, *RCT* Randomized Control Trial, *1°* primary intervention, *2°* secondary intervention

^b^calculated from values given in the Trial

**Table 6:** Effects of anti-ApoB therapy on LDL-related parameters^a^

Trial	Treatment	n	time (mo)	LDL-C				LDL-P				Non- HDL-C				ApoB			
				baseline	treatment	% dec	%tile	baseline	treatment	% dec	%tile	baseline	treatment	% dec	%tile	baseline	treatment	% dec	%tile
Cromwell [36]	Mip 200	83	6.5	NR	NR	–	–	1732	1208	30	32	NR	NR	–	–	NR	NR	–	–
[RCT;2°;FH, CHD]																			
Visser [37]	Mip 200	21	6.5	243	127	48	47	2207	1086	51	23	271	147	46	45	180	100	46	53
[RCT;2°;statin-intolerant]																			
		Total n	Mean time	Mean LDL-C				Mean LDL-P				Mean non-HDL-C				Mean ApoB			
				baseline	treatment	% dec	%tile	baseline	treatment	% dec	%tile	baseline	treatment	% dec	%tile	baseline	treatment	% dec	%tile
		104	6.5	243	127	48	47	1970 ± 336	1147 ± 86	41 ± 15	28 ± 6	271	147	46	45	180	100	44	53

Doses in mg/injection; *Mip* Mipomersen; *FH* familial hypercholesterolaemia; *CHD* coronary heart disease

^a^Month defined as 4 weeks, *LDL* low-density lipoprotein, *LDL-C* low-density lipoprotein cholesterol, *LDL-P* low-density lipoprotein particle number, *Non HDL-C* non-high-density lipoprotein cholesterol, *ApoB* apolipoprotein B, *% dec* percent decrease, *%tile* Percentile of Framingham population; *NR* not reported, *RCT* Randomized Control Trial, *1°* primary intervention, *2°* secondary intervention

**Table 7:** Effects of combination therapy on LDL-related parameters^a^

Trial	Treatment	n	time (mo)	LDL-C				LDL-P				Non- HDL-C				ApoB			
				baseline	treatment	% dec	%tile	baseline	treatment	% dec	%tile	baseline	treatment	% dec	%tile	baseline	treatment	% dec	%tile
Airan-Javia [20]	S 20 + ERN 2 g	26	12	123	75	39	2	1169	725	38	<2	151	92	39	3	97	61	37	4
[RCT;2°;carotid athero]																			
Berhanu [38]	PIO 30/45 + Statin	295	4.25	104	101	3	24	1527	1338	12	41	156^b^	135^b^	13	36	90	87	3	36
[RCT;2°;FH, CHD]																			
Jones [9]	LDS + F	146	3	NR	NR	–	–	2055	1246	39	35	NR	NR	–	–	NR	NR	–	–
[RCT;1°;mixed dyslipid]	MDS + F	154	3	NR	NR	–	–	1993	1149	42	28	NR	NR	–	–	NR	NR	–	–
Le [29]	E 10/S 10	160	6	156	72	54	2	1758	1090	38	23	192^b^	100^b^	48	9	152	90	40	40
[RCT;1°;dyslipid]	E 10/S 10 + N 2 g	293	6	156	63	60	2	1722	901	48	10	191^b^	84^b^	56	−3	151	77	49	24
Maki [11]	A10 + POM3	123	1	140	97	31	21	1971	1311	33	39	214	133	38	34	148	102	31	55
[RCT;1°;mixed dyslipid]																			
Goldberg[42, 56]	Colv 3.75 g + Met 1.7 g	145	4	129	101	22	24	1623	1336	18	41	168^c^	145^c^	14	43	109	96	12	48
[RCT;1°;T2D]																			
		Total n	Mean time	Mean LDL-C				Mean LDL-P				Mean non-HDL-C				Mean ApoB			
				baseline	treatment	% dec	%tile	baseline	treatment	% dec	%tile	baseline	treatment	% dec	%tile	baseline	treatment	% dec	%tile
		1342	5	135 ± 20	85 ± 17	35 ± 21	13 ± 12	1727 ± 293	1137 ± 224	34 ± 12	27 ± 15	179 ± 24	115 ± 26	35 ± 18	21 ± 20	125 ± 29	86 ± 15	29 ± 18	35 ± 18

Doses in mg or g/day; *S* Simvastatin; *ERN* Extended release niacin; *PIO* Pioglitazone; *LDS* Low-dose statin; *MDS* Moderate-dose statin; *F* Fenofibrate; *E* ezetimibe; *N* niacin; *A* Atorvastatin; *POM3* Prescription omega-3-fatty acid; *Colv* Colesevelam; *Met* Metformin; *carotidathero* carotid atherosclerosis; *FH* familial hypercholesterolaemia; *CHD* coronary heart disease; *mixed dyslipid* mixed dyslipidemia; *dyslipid* dyslipidemia;*T2D* type 2 diabetes; *met syn* metabolic syndrome

^a^Month defined as 4 weeks, *LDL* low-density lipoprotein, *LDL-C* low-density lipoprotein cholesterol, *LDL-P* low-density lipoprotein particle number, *Non HDL-C* non-high-density lipoprotein cholesterol, *ApoB* apolipoprotein B, *% dec* percent decrease, *%tile* Percentile of Framingham population; *NR* not reported, *RCT* Randomized Control Trial, *1°* primary intervention, *2°* secondary intervention

^b^calculated from values given in the Trial

^c^calculated using least squares mean

**Table 8:** Effects of anti-diabetic therapy on LDL-related parameters^a^

Trial	Treatment	n	time (mo)	LDL-C				LDL-P				Non-HDL-C				ApoB			
				baseline	treatment	% dec	%tile	baseline	treatment	% dec	%tile	baseline	treatment	% dec	%tile	baseline	treatment	% dec	%tile
GLAI [39]	PIO 30/45	333	6	107	120	−12	40	1394	1345	4	42	155^b^	159^b^	−3	54	NR	NR	–	–
[RCT;1°;T2D]	ROSI 4/8	325	6	109	130	−20	50	1368	1480	−8	51	153^b^	179^b^	−17	69	NR	NR	–	–
Goldberg [42, 56]	Met 1.7 g	141	4	136	129	5	49	1670	1551	7	56	173^c^	164^c^	5	58	112	107	4	61
[RCT;1°;T2D]																			
Shadid [40] [RCT;1°;obesity]	PIO 30	19	5	128	124	3	44	1420	1270	11	36	169^b^	154^b^	9	50	NR	NR	–	–
Szapary [41]	PIO 30/45	30	3	127	132	−4	51	1577	1428	9	48	170	174	−2	65	93	93	0	44
[RCT;1°;met syn]																			
		Total n	Mean time	Mean LDL-C				Mean LDL-P				Mean non-HDL-C				Mean ApoB			
				baseline	treatment	% dec	%tile	baseline	treatment	% dec	%tile	baseline	treatment	% dec	%tile	baseline	treatment	% dec	%tile
		848	5	121 ± 13	127 ± 5	5 ± 10	47 ± 4	1486 ± 131	1415 ± 110	4 ± 8	47 ± 8	164 ± 9	166 ± 10	2 ± 10	59 ± 8	103 ± 13	100 ± 10	2 ± 3	53 ± 12

Doses in mg or g/day; *PIO* Pioglitazone; *ROSI* Rosiglitazone; *Met*: Metformin; *T2D*: type 2 diabetes

^a^Month defined as 4 weeks, *LDL* low-density lipoprotein, *LDL-C* low-density lipoprotein cholesterol, *LDL-P* low-density lipoprotein particle number, *Non HDL-C*: non-high-density lipoprotein cholesterol, *ApoB* apolipoprotein B, *% dec* percent decrease, *%tile* Percentile of Framingham population; *NR* not reported, *RCT* Randomized Control Trial, *1°* primary intervention, *2°* secondary intervention

^b^calculated from values given in the Trial

^c^calculated using least squares mean

**Table 9:** Effects of other lipid-lowering therapies on LDL-related parameters^a^

Trial	Treatment	n	time (mo)	LDL-C				LDL-P				Non-HDL-C				ApoB			
				baseline	treatment	% dec	%tile	baseline	treatment	% dec	%tile	baseline	treatment	dec %	%tile	baseline	treatment	% dec	%tile
SANDS [43]	Aggressive	252	36	103	71	31	<2	1228	860	30	7	136	100	26	9	93	69	26	14
[RCT;1°;T2DM]	Standard	247	36	102	104	−2	27	1254	1159	8	28	137	138	−1	38	97	91	6	41
Ballantyne[44]	P 30 + Dal 300	76	3	95	102	−7	25	1275	1251	2	35	NR	NR	–	–	NR	NR	–	–
[RCT;1°;dyslipid]	P 30 + Dal 600	68	3	95	104	−9	27	1256	1220	3	33	NR	NR	–	–	NR	NR	–	–
	P 30 + Dal 900	74	3	93	97	−4	21	1244	1125	10	26	NR	NR	–	–	NR	NR	–	–
		Total n	Mean time	Mean LDL-C				Mean LDL-P				Mean non-HDL-C				Mean ApoB			
				baseline	treatment	% dec	%tile	baseline	treatment	% dec	%tile	baseline	treatment	% dec	%tile	baseline	treatment	% dec	%tile
		717	16	98 ± 5	96 ± 14	2 ± 17	20 ± 11	1251 ± 17	1123 ± 155	10 ± 11	26 ± 11	137 ± 1	119 ± 27	13 ± 19	24 ± 20	95 ± 3	80 ± 16	16 ± 14	28 ± 19

Doses in mg/day; Aggressive: Treatment to a LDL-C goal of ≤ 70 mg/dL and non-HDL-C goal of 100 mg/dL using a variety of treatment regimes including statins and exzetimibe; Standard: Treatment to a LDL-C goal of ≤ 100 mg/dL and non-HDL-C goal of 130 mg/dL using a variety of treatment regimes including statins and exzetimibe

*P* Pravastatin, *Dal* Dalcetrapib, *T2D* type 2 diabetes mellitus, *dyslipid* dyslipidemia

^a^Month defined as 4 weeks, *LDL* low-density lipoprotein, *LDL-C* low-density lipoprotein cholesterol, *LDL-P* low-density lipoprotein particle number, *Non HDL-C* non-high-density lipoprotein cholesterol, *ApoB* apolipoprotein B, *% dec* percent decrease, *%tile* Percentile of Framingham population; *NR* not reported, *RCT* Randomized Control Trial, *1°* primary intervention, *2°* secondary intervention

#### Statins

A total of 1,876 subjects were treated with statins for a mean of 4 months (range 1–12 months). Most subjects had hypercholesterolemia or mixed dyslipidemia with or without known CVD; two studies featured subjects with metabolic syndrome or diabetes. The types of statins studied included: atorvastatin (A) in 8 treatment arms [8–14], pravastatin (P) in 6 arms [13, 15–19], simvastatin (S) in 8 arms [9, 13, 20–22], rosuvastatin (R) in 3 arms [9, 12], and pitavastatin (Pit) in 1 arms [17]. The mean LDL-P and LDL-C concentrations at baseline were 1942 ± 380 nmol/L and 162 ± 32 mg/dL respectively (Table 2). The mean treatment-induced decrease in LDL-P across studies was 30 % producing an on-treatment level of 1346 ± 226 nmol/L which is equivalent to the 42nd percentile of the Framingham Offspring reference population. This was slightly less than the mean decrease in LDL-C (−34 %), which yielded an on-treatment level of 106 ± 27 mg/dL (29th percentile), and the mean decrease in non-HDL-C (−34 %, 35th percentile). The mean on-treatment apoB level was 103 ± 21 mg/dL (54th percentile) which represented a 27 % decrease from baseline. These results suggest that statins reduce LDL-C to a lower population percentile however, both LDL-P and apoB levels remained at elevated population percentiles signifying potentially higher residual cardiovascular (CV) risk.

#### Fibrates

Results from 7 studies of fibrate treatments involving 788 subjects with hypertriglyceridemia or known CVD are presented in Table 3. These included 4 studies with fenofibrate (F) [9, 21, 23, 24], 2 with bezafibrate (Bz) [25, 26] and one with gemfibrozil (G) [4]. Unlike statins, fibrates were found to increase LDL-C in most studies (mean +6 %) whereas LDL-P, non-HDL-C and apoB were reduced in most fibrate studies (mean decreases of 10 %, 14 % and 6 % respectively). The average population percentiles achieved for LDL-C, non-HDL-C and apoB were 44 %, 54 %, and 56 %, respectively. In contrast, the average on-treatment percentile achieved for LDL-P was 57 %. This highlights distinct differences in these biomarkers representing LDL-related risk for CVD.

#### Niacin

Niacin was evaluated in a total of 580 subjects enrolled across 6 studies (Table 4) [10, 27–33]. Baseline LDL-P and LDL-C levels were quite variable ranging from 1033 to 2561 nmol/L for LDL-P and 76 to 211 mg/dL for LDL-C. Small decreases (≤ 20 %) were observed in all LDL-related parameters with treatment. The mean population percentiles achieved on-treatment for LDL-P and LDL-C were 51 % and 53 % however in 2 studies, population percentiles for LDL-P of below 20 % were achieved [28, 31].

#### Bile acid Sequestrants

The bile acid Sequestrants colesevelam (Colv) and colestimide (Cole) were evaluated in 2 studies involving a total of 139 hypercholesterolemic subjects (Table 5) [34, 35]. Baseline LDL-P and LDL-C mean levels were significantly elevated (2206 ± 164 nmol/L and 178 ± 13 mg/dL, respectively). Both levels were decreased 9 % on treatment.

#### Anti-ApoB Oligonucleotides

Two studies reported results of treatment with the experimental injectable anti-apoB oligonucleotide mipomersin (Table 6) [36, 37]. One study was conducted in patients with familial hypercholesterolemia [36]; the other in statin-intolerant patients with high CVD risk [37]. Significant decreases in all LDL-related parameters were reported (mean −41 to −48 %). The average population percentiles achieved for LDL-C, non-HDL-C and apoB were 47 %, 45 %, and 53 %, respectively. In contrast, the average on-treatment percentile achieved for LDL-P was 28 %. Thus, LDL-P goals were closer to being achieved with treatment than LDL-C, non-HDL-C and apoB goals.

#### Combination Therapies

Therapies involving combinations of statins with one or more lipid lowering agent including niacin [20, 29], fenofibrate [9], ezetimibe [29] or prescription omega-3-fatty acid [11], pioglitazone [38], were evaluated in 1342 subjects across 6 studies (Table 7). On treatment, LDL-P and LDL-C were reduced 34 % and 35 %, to levels equivalent to the 27th and 13th percentiles of the reference population, respectively. Similar reductions in non-HDL-C (21 %) and apoB (35 %) were also observed. The most effective therapy was the combination of simvastatin with ezetimibe and niacin which decreased LDL-P and LDL-C by 48 % and 60 %, levels equivalent to the 10th and 2nd population percentiles, respectively [29].

#### Anti-diabetic agents

Results from 4 studies evaluating anti-diabetic therapies in subjects with metabolic syndrome or type 2 diabetes mellitus (T2D) are presented in Table 8. These included pioglitazone (3 treatment arms) [39–41], rosiglitazone (1 arm) [39] and metformin (1 arm) [42]. Overall, mean reductions in LDL-P and LDL-C were small (4–5 %).

#### Other lipid-lowering therapies

Two studies reported results from lipid-lowering therapies that could not be classified in the aforementioned treatment groups (Table 9). These included the Stop Atherosclerosis in Native Diabetics Study (SANDS) [43] and a study evaluating the CETP inhibitor dalcetrapib [44]. SANDS compared the effects of aggressive treatment to LDL-C and non-HDL-C targets vs. standard treatment. The aggressive treatment, consisting of statins, alone or in combination with other agents, decreased LDL-C and LDL-P to desired population percentile goals (<2 % and 7 %, respectively) whereas the standard treatment had little effect. In the dalcetrapib study in which subjects had LDL-C < 100 mg/dL but elevated LDL-P levels at baseline, treatment at the highest dose decreased LDL-P 10 % to a level equivalent to the 26^th^ population percentile.

### Medication, LDL-C and LDL-P Goals

Each study was evaluated to determine whether the following treatment goals recommended for high risk patients were met [45]: LDL-P ≤ 1000 nmol/L and LDL-C < 100 mg/dL. In the statin monotherapy studies, LDL-P goals were met in 4 % (1/22) of study arms and LDL-C goals met in 53 % (9/17) of study arms in which patients were above goal at initiation of treatment. In the niacin treatment group, LDL-P and LDL-C goals were met in 29 % (2/7) and 33 % (2/6) of study arms, respectively. In the statin combination therapy treatment group, LDL-P goals were met in 28 % (2/8) of study arms and LDL-C goals were met in 67 % (4/6) of treatment arms in which patients were above goal at initiation of treatment. In addition, LDL-C and LDL-P goals were met with aggressive, but not standard treatment, in SANDS. None of the other lipid-lowering therapies resulted in the attainment of LDL-P and LDL-C goals.

**Fig. 2:**
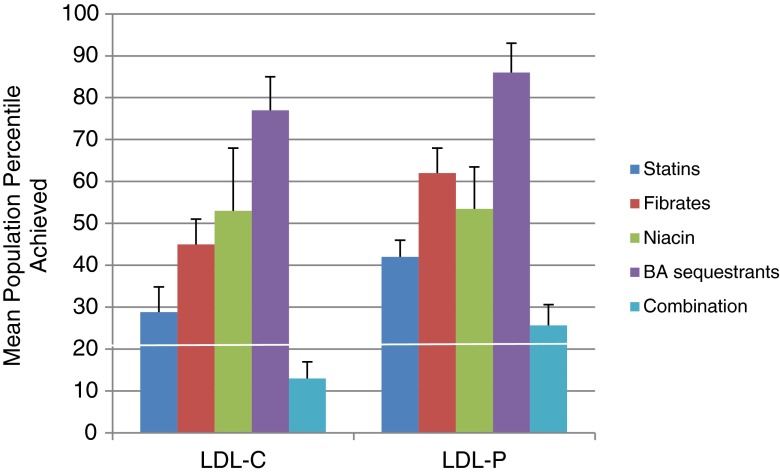
Percentile population achieved with therapy. The mean percentile of the reference population achieved with each treatment is shown. (+/− SEM)

Furthermore, as illustrated in Fig. 2, these responses are expressed in terms of percentile of the population achieved for each lipid lowering therapy. Various treatment options (except niacin) reduced LDL-C to a lower population percentile versus LDL-P suggesting possible residual CV risk with elevated LDL-P levels.

### Correlation of Lipid Parameters and CVD Progression

Three studies examined correlations between treatment-induced changes in lipid parameters and atherosclerosis progression [25, 43, 46] and one study examined correlations between changes in lipid parameters and new coronary events [4].

In the Pravastatin Limitation of Atherosclerosis in the Coronary Arteries (PLAC-I) Trial, 241 patients with angiographically defined coronary artery disease were randomized to treatment with 40 mg/day pravastatin or placebo [46]. Statin-treated subjects showed a 25 % decrease in LDL-P and 26 % decrease in LDL-C over 6 months and a significantly slower rate of lumen narrowing over 3 years compared to placebo controls (−0.018 vs. −0.053 mm/year, *p* < 0.01). Correlation of 6-month lipid levels and atherosclerosis progression showed that lumen narrowing was associated with higher on-study LDL-P (*r* = −0.13), independent of on-study LDL-C, HDL-C, and triglycerides.

In SANDS, 418 Native Americans with diabetes and no previous cardiovascular events were randomized to 1) aggressive statin treatment to targets of LDL-C ≤70 mg/dL, non-HDL-C ≤100 mg/dL, and systolic blood pressure (SBP) ≤115 mm Hg or 2) standard statin treatment to targets of LDL-C ≤100 mg/dL, non-HDL-C ≤130 mg/dL, and SBP ≤130 mm Hg [43]. CIMT was assessed as a measure of the severity of atherosclerosis. Most patients had been treated previously and had achieved standard targets at baseline (mean LDL-C of 103 and 102 mg/dL, mean non-HDL-C of 136 and 137 mg/dL, and mean SBP of 128 and 132 mm Hg for the aggressive and standard groups, respectively). After 36 months of treatment, the aggressive group had achieved significantly greater reductions in LDL-P (−30 % vs. −8 %), LDL-C (−31 % vs. +2 %), non-HDL-C (−26 % vs. +1 %), apoB (−26 % vs. −6 %) and a significant reduction in lumen narrowing (−0.020 vs. +0.038 mm) compared to the standard group. A comparison of changes in CIMT between the two groups, stratified by baseline lipid levels, showed no significant interactions between treatment and initial levels of LDL-P, LDL-C, non-HDL-C, HDL-C, triglycerides, or apoB indicating that the treatment effect did not differ by baseline levels of the measured lipid parameters. Further analysis found that changes in LDL-C and non-HDL-C were both independently correlated with CIMT regression, while changes in LDL-P and apoB showed borderline significance to CIMT regression at 36 months.

In the Veterans Affairs High-Density Lipoprotein Intervention Trial (VA-HIT), 1061 men with a diagnosis of established coronary heart disease (CHD) were randomized to 1200 mg/day gemfibrozil or placebo and treated for a median of 5.1 years. On-study lipid levels, obtained at the 7-month visit, showed significant increases in HDL-C (+6 % vs. 0 %) and HDL-P (+10 % vs. +6 %) and significant decreases in triglycerides (−30 % vs. +6 %), non-HDL-C (−5 % vs. +1 %), apoB (−6 % vs. −3 %), and LDL-P (−5 % vs. +7 %) for the gemfibrozil group compared to the placebo group [4]. Among those patients who had a coronary event, baseline and treatment levels of LDL-P were strong independent predictors of a new coronary event, while neither baseline nor on-study levels of LDL-C, HDL-C, or triglycerides were significant predictors of CHD risk.

## Discussion

The objective of this analysis was to determine the effect of different LDL-lowering therapies on various LDL markers. Study-level correlations between treatment-related changes in lipid parameters and CVD progression were reported, as well as the population percentile achieved based on data from the Framingham Offspring Study. Among the lipid-lowering monotherapies studied, the anti-apoB therapy (mipomersen) and the statins produced the greatest reductions in LDL-P and LDL-C, followed by niacin, fibrates and bile acid sequestrants. Furthermore, the combination of statins with other agents led to greater reductions in LDL-P and LDL-C than the statins alone. Although the combination of fibrates with statins generally decrease LDL-C and LDL-P levels (Table 7), the effect can vary and increases in these parameters have been observed in certain populations [47]. Studies evaluating omega-3-fatty acids as monotherapy were beyond the focus of this review however, a study was published reporting the effects of a pure form of icosapenty ethyl eicosapentaenoic acid (IPE) on lipoprotein particles in hypertiglyceridemic patients. This study showed that in subjects treated with 4 g/day IPE for 12 weeks, changes in LDL-P (−0.1 %) and LDL-C (−6.5 %) from baseline were negligible [48].

Therapies targeting LDL-C are easily evaluated in routine clinical practice because of the widespread availability of LDL-C measurements; LDL-C is most commonly estimated from measurements of total cholesterol using the Friedewald formula. However, as illustrated in this analysis, various treatment options demonstrated variable effects on LDL-C relative to LDL-P. Study-level data from this systematic review showed that statins generally lower LDL-C more than they lower LDL-P, whereas fibrates lower LDL-P more than they lower LDL-C. Therefore, reliance on only LDL-C as a biomarker of LDL-related risk of CVD may not fully appreciate the benefit of these therapies due to the fact that LDL-C and LDL-P may not agree at times and are considered discordant measures.

Why is there discordance between LDL-C and LDL-P?The cholesterol content of LDL particles varies between individuals owing to differences in the relative content of cholesteryl ester and triglycerides within the core of LDL particles [3, 49]. The majority of atherogenic lipoproteins in individuals with insulin resistance, metabolic syndrome or T2D are smaller, cholesterol-depleted LDL particles. These compositional changes in LDL particles may lead to a disagreement between measures of LDL-C and LDL-P resulting in “discordance” [2]. As demonstrated in this analysis, statins tend to lower LDL-C more than LDL-P, hence creating discordance whereby LDL-C targets may be achieved while LDL-P levels remain elevated. This indicates the potential for residual CVD risk.

The clinical consequences of discordant measures of LDL-P and LDL-C have been investigated in MESA [2] and the Framingham Offspring Study [1]. In both studies, in individuals with discordant LDL-P and LDL-C levels, it was LDL-P and not LDL-C that tracked well with CVD risk.

This systematic review also identified 2 randomized controlled trials that reported correlations between lipid and lipoprotein parameters and CVD progression. A strong correlation between on-study LDL-P and CVD progression was observed in the PLAC-1 trial which found that lumen narrowing was associated with higher on-study LDL-P, independent of LDL-C, HDL-C, and triglycerides [46]. In addition, the VA-HIT study found that on-study LDL-P was a strong, independent predictor of new coronary events, while on-study LDL-C, HDL-C, and triglycerides were not significant predictors of CVD risk [4]. Overall, these results are consistent with study-level analyses from several large epidemiologic studies, including the EPIC-Norfolk [50, 51], Framingham Offspring [1], MESA [2], and Women’s Health studies [5, 15], which found that LDL-P was a better discriminator of cardiovascular risk than LDL-C.

## Lipoprotein Management

Measurement of LDL-P levels is recommended for high-risk patients whose LDL-C levels are optimal, near optimal or borderline-high, but who also have additional CHD risk equivalents. These include patients with CVD, metabolic syndrome or T2D that are near or at a LDL-C goal of >70 mg/dL and <100 mg/dL; or patients with metabolic syndrome, low HDL-C levels or T2D near or at a goal of LDL-C <100 mg/dL. What therapeutic targets should be established for apoB, LDL-C, non-HDL-C, and LDL-P in these patients? The American Association of Clinical Chemistry (AACC) suggest the following goals: apoB <80 mg/dL, LDL-C <100 mg/dL, non-HDL-C <120 mg/dL and LDL-P <1100 nmol/L [6, 52]. When a patient is at or near their LDL-C goal with discordantly high LDL-P, then one can conclude that the patient has residual LDL-related risk and more intense LDL lowering therapy is needed. Conversely, when LDL-P levels are within the target range, then current therapy is adequate [3]. Therefore, the information gleaned from the LDL-P concentration should be utilized to aid clinical decision-making regarding drug choice and dosing. This can be especially helpful with regard to combination therapies which differentially affect LDL-C and LDL-P [44].

## Perspectives and Future Directions

In this study, we conducted a systematic review that reports the effects of multiple classes of lipid modifying agents on LDL-C and LDL-P. In the future, new classes of LDL-C lowering therapies will further challenge the use of the Friedewald formula to calculate LDL-C levels. Certain medications that inhibit CETP [53] and proprotein convertase subtilisin/kexin type 9 (PCSK9) [54] have reported limitations of the LDL-C estimation by the Friedewald formula, which overestimates the LDL-C lowering effect of these agents when compared to standardized ultracentrifugation methods (beta quantification). These obstacles may be overcome by measuring LDL-P and/or apoB.

LDL-P measurements provide more accurate information concerning risk stratification in most studies, and a potential target to guide adjustment for LDL-C lowering therapy. Among subjects with LDL-C at their minimal acceptable goal, an elevated LDL-P would confer risk that may be reduced by adjustment of the statin dosage, change in statin from a less efficacious to more efficacious agent, or the addition of a further LDL-P lowering agent. Contemporary lipid lowering management practices tend to overtreat with statins, particularly for intermediate and high risk patients, to LDL-C values that are pushing beyond 70 mg/dL. The use of LDL-P as a management target in the presence of discordantly high LDL-P values can individualize aggressive lipid-lowering therapy and in the process potentially reduce statin intolerance and be more cost-efficient. In addition, patients with LDL-C above their minimal acceptable target who have low LDL-P may not need intensification of their cholesterol lowering therapies.
